# Editorial: Dual disorders (addictive and concomitant psychiatric disorders): Mechanisms and treatment

**DOI:** 10.3389/fpsyt.2022.975674

**Published:** 2022-08-02

**Authors:** Florence Vorspan, Georges Brousse, Wim Van Den Brink

**Affiliations:** ^1^Université de Paris Cité, UFR de Médecine, INSERM UMRS1144, Paris, France; ^2^Assistance Publique – Hôpitaux de Paris, GHU.NORD, Hôpital Fernand Widal, Paris, France; ^3^Université Clermont Auvergne, CNRS Institut Pascal, Clermont Auvergne INP, Clermont-Ferrand, France; ^4^Centre Hospitalier Universitaire de Clermont-Ferrand, Clermont-Ferrand, France; ^5^Service D'addictologie, Amsterdam University Medical Centers, Location Academic Medical Center, University of Amsterdam, Amsterdam, Netherlands

**Keywords:** comorbidity, co-occurrence, dual disorders, psychiatry, addiction, pathophysiology, association

When we launched this Research Topic dedicated to “*Dual Disorders: Mechanisms and Treatment*” we were highly ambitious. We wanted to offer the opportunity to colleagues all over the world to use it as a window to show their latest research findings. We were especially eager to read and publish new empirical evidence on the nature of the relationship between addiction and other psychiatric disorders as well as new empirical evidence on the treatment of dual disorders.

Indeed, we already know since several decades, that dual disorders, i.e., the comorbidity between addictive and other psychiatric disorders, are the rule rather than the exception. The high prevalence of dual disorders and their association with worse outcomes, not only related to poor compliance, are already well-documented.

The debate on the mechanisms leading to dual disorders as either the result of a self-medication by psychiatric patients, the result of repetitive substance use toxicity on brain functions such as mood dysregulation, or the result of some shared biological (e.g., genetic) or environmental (e.g., childhood adversity) factor, will not be solved by this Research Topic, but the 12 articles published are a good reflection of current researchers' concerns.

Two published articles from this Research Topic are literature reviews. The first one is a general review on how Research Domain Criteria (RDOC) could serve as a basis of dual disorders research (Hakak-Zargar et al. from Canada) taking examples in several specific dual disorders. The second one is dedicated to one dual disorder: the co-occurrence a Post-Traumatic Stress Disorder (PTSD) and one or several addictive disorders (Renaud et al. from France). The authors have read the literature with a specific focus on the mechanisms linking PTSD symptoms and craving, trying to identify a mechanism behind the worse prognosis of addictions in Substance Use Disorders (SUD) patients *with* compared to SUD patients *without* PTSD.

There are also ten studies with original data published in this Research Topic. Three are cross-sectional studies conducted in the general population, exploring potential mechanisms causing dual disorders. Bourduge et al. from France, explored through questionnaires the association between the first lockdown in French teenagers, coping strategies and substance use, as a model of adaptation disorders. Ágoston et al. in a collaborative work conducted between Hungary and the Netherlands, observed the link between a higher score to a caffeine dependence screening scale and a higher score to adult Attention Deficit/Hyperactivity Disorders (ADHD) screening score, that can serve for a model of the association of stimulant abuse and adult ADHD. Finally, El Archi et al. from France, conducted an internet survey showing the link between a screening questionnaire of gambling disorder and a screening score of adult ADHD, but also depressive symptoms.

The last seven studies, all conducted in patient samples using various methodologies. Three of them were cross-sectional descriptive studies.

Cabé et al. from France, showed a significant association between symptoms of a “high” during cocaine use and the self-report of depression during cocaine “downs.” Icick et al. in a collaborative study comparing bipolar patients treated in expert centers in France and Norway, observed statistically different prescribed treatments according to the presence of specific SUDs (cannabis, alcohol, or tobacco use disorder). Lastly, Barrangou-Poueys-Darlas et al. from France, descripted the prevalence of a high score on ADHD screening scales and anxiety disorders in patients in care for Gambling Disorders.

Four prospective experimental studies conducted in patients open an avenue for intervention studies in patients with dual disorders.

Therribout et al. from France, describe their stringent methodology to assess ADHD diagnosis in patients with severe SUD. Cardullo et al. from Italy, conducted a secondary analysis of a prospective r-TMS trial comparing cocaine use disorder patients with and without comorbid ADHD. They did not show a difference in the treatment response between the two groups. Todesco et al. from Canada, studied the predictive power of a decision-making test among treatment seeking dual disorder patients, showing that 4 dimensions of this test predicted drop-out in these patients. Lastly, Fonseca et al. from Spain, prospectively (90 days) studied patients with a major depression with and without cocaine use disorder, assessing cortisol and BDNF levels. Their results suggest that the combination of cortisol and BDNF plasmatic levels could differentiate primary vs. cocaine-induced major depression.

This variety of articles show that dual disorders research is moving forward. On the one hand research involves more and more specific association of pairs of psychiatric and addictive disorders, and on the other hand recent research tries to better understand the mechanisms behind the occurrence or severity of dual disorders. Specific therapeutic studies matching treatments with certain patient characteristics are at reach. We hope that reading those articles will give you plenty of new ideas to move this field forward. Patients suffering from dual disorders are still in great need of effective treatments, and high quality research aiming at changing the poor prognosis of these co-occurring conditions is warranted.

## Author contributions

All authors listed have made a substantial, direct, and intellectual contribution to the work and approved it for publication.

## Conflict of interest

The authors declare that the research was conducted in the absence of any commercial or financial relationships that could be construed as a potential conflict of interest.

## Publisher's note

All claims expressed in this article are solely those of the authors and do not necessarily represent those of their affiliated organizations, or those of the publisher, the editors and the reviewers. Any product that may be evaluated in this article, or claim that may be made by its manufacturer, is not guaranteed or endorsed by the publisher.

